# The effect of financial goal pursuit on trust and trustworthiness among Chinese college students: Demographic differentials

**DOI:** 10.1186/s40359-025-03274-y

**Published:** 2025-08-12

**Authors:** Xinyi Wei, Kaiji Zhou, Huiling Zhou, Jiayi Jiang, Lei Ren, Pengcheng Wang, Chang Liu, Lin Lu, Caiyu Wang, Jingyu Geng

**Affiliations:** 1 School of Nursing, Putian University, Putian, 351100 China; 2 College of Psychology, Sichuan Normal University, Chengdu, 611100 China; 3 Department of Applied Social Sciences, The Hong Kong Polytechnic University, Hong Kong, China; 4 School of Psychology, Shanghai Normal University, Shanghai, 200234 China; 5 Department of Psychology, Renmin University of China, Beijing, 100872 China; 6 Military Psychology Section, Logistics University of PAP, Tianjin, 300309 China; 7 School of Media and Communication, Shanghai Jiao Tong University, Shanghai, 200240 China; 8 BrainPark, School of Psychological Sciences, Turner Institute for Brain and Mental Health, Monash University, Clayton, VIC 3800 Australia; 9 School of Business Administration, Southwestern University of Finance and Economics, Chengdu, 611100 China; 10 School of Teacher Education, Shaoxing University, Shaoxing, 312000 China; 11 Department of Psychology, Normal College, Qingdao University, Qingdao, 266071 China

**Keywords:** Financial success, Interpersonal trust, University students, Gender, Origin

## Abstract

**Background:**

College students’ financial goal pursuit might have profound impacts on both individuals and society. Despite the well-established research on the impacts of financial goal pursuit on individuals’ well-being, direct investigations into its effects on trust and trustworthiness among college students are relatively limited, especially in the Chinese context. Moreover, studies explored individual variations that might moderate the effects of financial goal pursuit on trust and trustworthiness are rare.

**Methods:**

This study examined the relationships between financial goal pursuit and trust and trustworthiness via an online survey (Study 1) and an experiment (Study 2) among 697 Chinese college students (289 in Study 1, 408 in Study 2) and explored the moderating roles of demographic variables, including gender, origin (urban versus rural), age, and family income. Financial goal pursuit was measured by Aspiration Index-6 in Study 1 and activated by images of luxury goods in Study 2. Trust and trustworthiness were measured by the classic investment game in both studies.

**Results:**

Study 1 showed significant negative associations between dispositional financial goal pursuit and trust and trustworthiness. These associations are only observed among male and urban students, with no significant associations found for female and rural students. Study 2 showed that activating financial goal pursuit could reduce trust and trustworthiness. Notably, the adverse effect on trust (but not trustworthiness) is more conspicuous among male and urban students.

**Conclusion:**

This research accentuates the necessity for nuanced understanding in the realm of financial pursuits, interpersonal trust, and demographic variables, especially in rapidly evolving socio-economic contexts like China.

## Introduction

The financial goal pursuit, as defined by Kasser and Ryan, refers to the aspiration and determination to achieve financial success and material wealth [1]. This concept is based on their broader theory of human motivation, which posits that people can have different types of goals, ranging from intrinsic goals, such as personal growth and community feeling, to extrinsic goals, like financial success and popularity.

Among university students, financial goal pursuit becomes particularly relevant. As students grow up from adolescence to adulthood, they start to formulate their values and personal/professional goals, among which the pursuit of financial success may become a priority. In addition, compared with younger students (e.g., middle school students), college students typically have higher autonomy in consumption and goal setting while facing greater consumer temptations, rendering them more likely to go astray in their pursuit of financial goals. This is particularly true in societies like China, where rapid economic growth has led to increased opportunities for wealth accumulation, and social norms place a high emphasis on financial success [2]. A global survey by Ipsos showed that over 70% of Chinese respondents assess their success based on the wealth they own, ranking first among all surveyed countries [3]. Thus, a focus on financial goal pursuit among Chinese college students raises concerns, as numerous previous studies have revealed that excessive focus on extrinsic goals, including financial success, can lead to various negative psychological outcomes, such as mental health issues and lower subjective well-being [4]. Nevertheless, compared to the well-documented influence of financial goal pursuit on well-being, research on its impacts on trust and trustworthiness is limited, especially in the Chinese context.

Trust refers to a psychological state in which an individual, based on positive expectations of others’ intentions and actions, willingly places their interests in a position vulnerable to harm by others [5]. Trustworthiness refers to the characteristics of integrity, honesty, and being worthy of trust [6]. That is, trust refers to how credible an individual perceives others to be, while trustworthiness refers to how deserving an individual is of others’ trust. For example, Trust can be demonstrated by a college student who believes that their roommate will not betray their trust, and thus decides to entrust the dormitory funds to the roommate for safekeeping. Trustworthiness is exemplified by the college student who, after taking on the responsibility of managing the dormitory funds, consistently keeps them safe, thereby proving to be worthy of the trust placed in them by the other roommates. 

Delineating how financial goal pursuit influences trust and trustworthiness within the context of China could contribute to the literature in this field for cultural, socioeconomic, and practical reasons. First, Western scholars have often regarded China as a collectivistic-oriented country where ingroup harmony is valued [7]. The cultural background divergence implies disparities between the current research and previous studies conducted within Western individualistic contexts. Second, trust and trustworthiness are fundamental elements of social cohesion and stability [8, 9] and are crucial in collaboration and teamwork, which are key for many professional settings [10, 11]. In China, trust significantly contributes to economic performance [12]. Other studies have repeatedly shown that interpersonal trust positively affects economic development, social life, and individual well-being [13–15]. However, some studies indicate that in recent years, with China’s marketization process, levels of trust have continuously declined [16, 17]. Third, the current investigation could benefit policymakers in China and other developing countries. Many developing countries, including Vietnam, have drawn inspiration for their market system reforms from China’s experience, but they may not notice that a decline in interpersonal trust could be one of the psychological costs or consequences of marketization, a consideration that should also concern other developing countries in the midst of marketization. Financial goal pursuit is regarded as one of the individual-level consequences of marketization [18]. Exploring the potential impact of financial goal pursuit on trust and trustworthiness in China can provide implications for other developing countries.

Theoretically, pursuing financial goals might undermine both trust and trustworthiness. First, financial success emphasizes self-interest and competition, which are the opposite of pro-social values, such as meaningful relationships and benevolence, according to the value system theories [19, 20]. This can undermine individuals’ trust in others by affecting their interpersonal relationships, which are a key source of trust. For instance, prioritizing financial success might lead individuals to choose friends based on their utility rather than their personal qualities, which could undermine the quality and trustworthiness of relationships [21]. Meanwhile, excessive financial goal pursuit may lead to reduced prosocial behaviors, increasing the likelihood of compromising ethical standards and making unethical decisions. This can undermine integrity, honesty, and reliability [21], thus dampening their trustworthiness.

A few empirical studies have associated emphasis on financial success with reduced trust and trustworthiness. Rahn & Transue [22], based on a large-scale survey, found that emphasis on wealth is negatively correlated with American adolescents’ social trust. In the game task involving resource scarcity in Bauer et al.’s experiment [23], American university students in consumer cuing condition (which is regarded as activating the desire for wealth) tend to exhibit higher levels of greed and behaviors betraying others (low trustworthiness) than those in the control condition. At the same time, they also tend to display a propensity to worry about being betrayed by others (low trust). Therefore, we hypothesize that the pursuit of financial goals can also undermine trust and trustworthiness among Chinese college students.

Previous cross-temporal meta-analyses have revealed a significant downward trend in the change of Chinese college students’ levels of trust [24]. Such a decline in trust is concerning, given the critical roles of trust in society and personal life. Furthermore, a decline in trust likely accompanies a corresponding change in collective trustworthiness. As trust is the perception of others’ trustworthiness, one’s declined trust may imply the reduced trustworthiness of people around. Indeed, a survey conducted among college students in China showed that more than two-thirds of the respondents stated their involvement in dishonesty in examinations and assignments at least once during their previous academic year [25], possibly implying a concerning prevalence of untrustworthiness in Chinese college students. Examining the possibly important role played by financial goal pursuit in Chinese college students’ trust and trustworthiness helps better understand these circumstances, providing evidence and implications for future education and administration practice.

Meanwhile, there is a noticeable gap in existing literature regarding the individual characteristics that might influence the relationship between financial goal pursuit and trust behaviors among college students. This research addresses this deficiency by not only examining the main effect of financial goals on trust and trustworthiness but also identifying when and for whom these effects are most pronounced. Such insights are essential for developing targeted interventions or administration strategies. Our study specifically focuses on the potential moderating effects of several commonly considered demographic variables in studies among Chinese students, including gender, student origin (urban versus rural), age, and family income, offering a detailed exploration of how these factors may influence trust dynamics.

Parental investment theory and social role theory predict that women trust less than men due to a higher sensitivity to risk and betrayal, while men trust more than women to maximize resources [26]. Following this logic, such a tendency of men in the trust situation may alter (or even reverse) the adverse effect of financial goal pursuit on trust, as the financial goal might lead men to be more inclined to ‘maximize resources’ through trust (e.g., in the trust game paradigm where the trustor invests money in the trustee to make profits) [27]. Indeed, empirical studies suggest that men trust more than women [26, 28]. However, contrasting studies have found either higher levels of trust in women compared to men or no significant gender differences at all [29]. Similar mixed patterns of findings are also observed for trustworthiness [29, 30]. Whether or how gender alters the influence of financial goal pursuit remains unknown.

Student origin may influence the pursuit of financial goals. Urban areas are commonly more commercialized than rural areas after decades of growth of the city-centered commodity economy in China. Dittmar’s consumer culture impact model [31] predicts such a commercialized social context exposes people to excessive consumerism messages via advertising, media, and peers, leading to the internalization of the consumer culture and materialism (a value orientation emphasizing financial success) [2]. The more internalized financial goal in students from urban areas may exert a more paramount impact on trust and trustworthiness. Nevertheless, the advanced market economy in urban areas may also lead to the internalization of the ethics of commercial society, including integrity and spirit of contract, which emphasizes the ethical pursuit of financial goals, potentially counteracting the negative effects on trust and trustworthiness. However, there is an absence of clear empirical evidence showing how it may alter (amplify or mitigate) the influence of financial goal pursuit.

Past research has associated age with trust. For example, Sutter and Kocher [32] found that trust increases almost linearly from early childhood to early adulthood. Meanwhile, Matsumoto et al. found that prosocial behavior increases with age, which may affect trustworthiness [33]. However, since our sample exclusively consists of college students—essentially a homogeneous group of young adults—it might be challenging to detect an age effect due to the limited age range.

As for students’ family income, past research suggests that lower-class individuals are more generous, trusting, charitable, and helpful compared with their upper-class counterparts [34], which might, in turn, mitigate the negative effect of financial goal pursuit on trust and trustworthiness of students from lower-income families. However, more recent research did not find evidence supporting this effect, including two high-powered and preregistered replication studies [35]. How income may interplay with financial goal pursuit in predicting trust and trustworthiness is unknown.

In sum, although a few empirical studies have suggested the link between the emphasis on the goal of financial success and reduced trust and trustworthiness, empirical evidence from Chinese college students is rare. Additionally, the interaction between college students’ demographic characteristics and financial goal pursuit remains an area yet to be clarified.

## The present research

This research aims to investigate two research questions: First, how does financial goal pursuit impact trust and trustworthiness among Chinese college students? Second, do demographic characteristics such as gender, origin, age, and family income influence the relationship between financial goal pursuit and trust and trustworthiness outcomes?

To address these questions, our study is divided into two parts. Study 1 examines the correlation between dispositional financial goal pursuit and trust, as well as trustworthiness, to establish baseline associations. Study 2 builds on these findings by exploring the effects of situational financial goal pursuit, activated through experimental manipulation, to assess causality.

Our hypotheses are as follows: dispositional financial goal pursuit is negatively correlated with both trust (Hypothesis 1a) and trustworthiness (Hypothesis 1b). We hypothesize that situational financial goal pursuit similarly undermines trust (Hypothesis 2a) and trustworthiness (Hypothesis 2b). Additionally, this research includes exploratory examinations of how gender, origin, age, and family income may moderate these relationships in both studies, aiming to uncover variations in these effects across different demographic settings.

In addition, in our Study 2, as the activation of financial goal pursuit may cause a rise of pleasure [15], we examined participants’ pleasure emotion during the experiment and controlled for it in statistical analyses to rule out its potential effects on participants’ responses to outcome measures; furthermore, we examined the interaction effects between the experimental condition (activation versus control) and participants’ feelings of pleasure.

### Study 1

#### Methods

##### Power analysis and participants

The sample size was estimated using the G*Power 3.1.9.2 software. The statistical test type was selected as “Linear multiple regression: R²increase;” in the absence of prior studies with identical designs, the effect size was set at the medium level of 0.15 for regression analysis; the alpha (α) level was set at 0.05, and the power was set at 0.95. The number of predictors, including the base variables and the interaction terms of interest in this study, was 9. Based on these parameters, the required sample size was calculated to be 166.

333 college students were selected from two universities in China. 289 participants provided valid data (89.5%). The mean age was 20.727 years (*SD* = 2.672), with 101 males, 188 females, 107 urban students, and 182 rural students. The academic year breakdown is 67 freshmen, 101 sophomores, 80 juniors, 24 seniors, 6 first-year graduate students, 9 second-year graduate students, and 2 third-year graduate students. In terms of family monthly income (in CNY), 60 students reported earnings below 3,000; 87 students between 3,000 and 5,000; 77 students between 5,001 and 10,000; 35 students between 10,001 and 15,000; 12 students between 15,001 and 20,000; 8 students between 20,001 and 30,000; 5 students between 30,001 and 50,000; 5 students between 50,001 and 100,000; and none reported more than 100,000. Formal informed consent was obtained from all participants.

## Materials and procedures

### Aspiration index (AI-6)

Financial goal pursuit was evaluated using the 6-item Aspiration Index (AI–6) developed by Sheldon & Kasser [36] and the Relative Financial Goal Importance (RFGI) method proposed by Ku, Dittmar, and Banerjee [37]. The AI–6 instrument comprises two dimensions: intrinsic (e.g., pursuing self-acceptance) and extrinsic goals (e.g., pursuing financial success), with three items each, scored on a 5-point scale. The extrinsic goals sub-scale had a Cronbach’s α of 0.700, the intrinsic goals subscale α was 0.640, and the overall α for both dimensions was 0.670. Financial goal pursuit scores were computed using the RFGI method. This method calculates RFGI by subtracting the overall average score of the scale from the financial aspiration score. Such a calculation reflects the priority of a specific goal in an individual’s goal system and captures the essence of financial goal pursuit more effectively [31]. A positive score indicates that financial goal pursuit surpasses overall goals in importance, while a negative score suggests the opposite. Higher scores indicate higher levels of financial goal pursuit.

### Investment game task

The classic investment game task was used to measure participants’ trust and trustworthiness [27, 38]. Participants were asked to assume that they had an initial amount of 100 CNY. Participants were paired with a hypothetical partner (also endowed with 100 CNY). Participants, acting as the trustor, had to decide how much money (n, 0 ≤ *n* ≤ 100) to give to the trustee. The trustee then received 3n and decided how much money (m, 0 ≤ m ≤ 3n) to return to the trustor. Final payoffs were 100 − n + m for the trustor and 100 + 3n − m for the trustee. Trust level was inferred from the amount invested by the trustor and the expected return rate [39]. Participants chose a number from 0 to 100 for their decisions. Later, trustworthiness was assessed by putting participants in the trustee’s position, imagining receiving 90 CNY from the investment of a trustor (who invested 30 CNY) and reporting the amount they would return (a number between 0 and 90).

## Procedures

Data collection was conducted via online surveys, with participants being informed that they were participating in a study examining the social behaviors of college students. After reading the instructions, participants first completed the AI-6, followed by the Investment Game Task. To ensure participants comprehended the rules of the investment game, control questions were inserted during the trustor and trustee scenarios. Only data from participants who answered the control questions correctly were analyzed. Finally, demographic information, including age, family income, gender, place of origin, and academic year (from first-year undergrad to third-year master’s), was collected.

## Results and discussion

Table 1 presents the descriptive statistics for participants’ perceived importance of financial objectives, the amounts they invested in the investment game (trust), their anticipated return ratios (trust), and the amounts they returned when acting as trustees (trustworthiness). Our findings of the trust and trustworthiness indicators (the proportion sent and returned around 50%) are generally comparable with the results of a meta-analysis of the investment game [40]. The results of the correlation analysis indicated significant relationships between the amounts invested, the anticipated return ratio (by the trustor), the amount returned (by the trustee), and the RFGI. There were also significant correlations between the amounts invested, anticipated return ratios, and amounts returned. Moreover, gender was correlated with RFGI.

**Table 1:** Descriptive statistics and correlation analysis (Study 1)

Variable	1	2	3	4	5	6	7	8
1. Gender	—							
2. Origin	0.069	—						
3. Age	-0.144^*^	-0.038	—					
4. Income	0.021	-0.008	-0.030	—				
5. Amount sent	-0.065	-0.094	-0.040	-0.005	—			
6. Estimated return rate	0.058	-0.033	-0.108	-0.024	0.475^***^	—		
7. Amount returned	0.078	-0.078	-0.048	0.046	0.327***	0.517***	—	
8. RFGI	0.084	0.116^*^	-0.064	-0.048	-0.182**	-0.187**	-0.242***	—
*M*			20.727	2.727	54.398	42.806	48.367	-0.491
*SD*			2.672	1.520	29.238	21.306	18.034	0.659

Note: RFGI = relative financial goal importance; * *p* < 0.05, ** *p* < 0.01,*** *p* < 0.001

In the regression analysis, demographic variables and RFGI served as independent variables, while the amount invested, anticipated return rate, and the amount returned by the trustees were treated as dependent variables, reflecting participants’ levels of trust and trustworthiness. In the first step of the regression, we introduced demographic variables and RFGI as independent variables into the model using the ‘Enter’ method. In the second step, interaction terms between RFGI and each demographic variable were added to the model in the same manner. Interactions that were found to be non-significant were excluded from the analyses. For regression analysis, dichotomous variables such as gender and place of origin were coded as 0 or 1, indicating female and rural as 0 and male and urban as 1, respectively.

After controlling for demographic variables, RFGI negatively predicted trust, indicated by the amount sent (B = -7.880, *p* = 0.003, Cohen’s *f*^2^ = 0.033) and the estimated return rate (B = -6.568, *p* < 0.001, Cohen’s *f*^2^ = 0.042). It also negatively predicted trustworthiness, as indexed by the amount returned (B = -8.764, *p* < 0.001, Cohen’s *f*^2^ = 0.033).

Table 2 shows that most interactions between RFGI and demographic variables (gender and origin) in predicting outcomes were significant, with the exception of the interaction between RFGI and gender regarding the amount sent, which was not significant. Specifically, the interaction between RFGI and origin was significant on amount sent (B = -12.257, *p* = 0.020, Cohen’s *f*^2^ = 0.019); the simple slope analysis showed that the link between RFGI and amount sent was significant among students from urban areas (B = -13.784, *p* < 0.001, Cohen’s *f*^2^ = 0.160) but was not significant among those from rural areas (B = -0.810, *p* = 0.830 Cohen’s *f*^2^ = 0.000). The interactions of RFGI with gender (B = -11.859, *p* = 0.003, Cohen’s *f*^2^ = 0.032) and origin (B = -8.005, *p* = 0.034, Cohen’s *f*^2^ = 0.016) were significant on estimated return rate; the negative relationship between RFGI and estimated return rate was significant among male students (B = -10.615, *p* < 0.001, Cohen’s *f*^2^ = 0.243) but not female students (B = 2.005, *p* = 0.561, Cohen’s *f*^2^ = 0.002), and it was significant among urban students (B = -10.867, *p* < 0.001, Cohen’s *f*^2^ = 0.168) but not rural students (B = -2.116, *p* = 0.427, Cohen’s *f*^2^ = 0.004).

**Table 2:** Multiple regression analyses with interactions (Study 1)

	Amount sent		Estimated return rate		Amount returned	
	B	95%CI	B	95%CI	B	95%CI
**Step 1**						
Gender	3.910	-3.215, 11.035	-2.678	-7.876, 2.519	-3.179	-7.533, 1.175
Age	-0.704	-1.975,0.567	-0.914	-1.842, 0.015	-0.360	-1.137, 0.417
Origin	-4.507	-11.592, 2.418	-0.223	-5.355, 4.909	-1.752	-6.032, 2.529
Income	-0.222	-2.431, 1.987	-0.535	-2.147, 1.078	0.385	-0.965, 1.735
RFGI	-7.880^**^	-13.015, -2.745	-6.568^***^	-10.308, -2.829	-8.764^***^	-9.902, -3.626
∆R^2^	0.047^*^		0.056^**^		0.074^**^	
**Step 2**						
RFGI*gender	—	—	-11.859^**^	-19.735, -3.983	-7.602^*^	-14.253, -0.851
RFGI*origin	-12.257^*^	-22.549,-1.966	-8.005^*^	-15.407, -0.602	-7.116^*^	-13.367, -0.365
∆R^2^	0.018^*^		0.045^**^		0.034^**^	

Note. Nonsignificant interaction terms of RFGI with age and income were excluded for simplicity; the interaction between RFGI and gender on the amount sent was nonsignificant, thus also being excluded

For the amount returned, significant interactions were observed between RFGI and gender (B = -7.602, *p* = 0.025, Cohen’s *f*^2^ = 0.018), as well as between RFGI and origin (B = -7.116, *p* = 0.034, Cohen’s *f*^2^ = 0.016); the relationship between RFGI and amount returned was significant among male students (B = -9.878 *p* < 0.001 Cohen’s *f*^2^ = 0.319) but was not significant among female student (B = -1.092, *p* = 0.707, Cohen’s *f*^2^ = 0.001), and it was significant among urban students (B = -10.644, *p* < 0.001, Cohen’s *f*^2^ = 0.248) but not rural students (B = -2.892, *p* = 0.215, Cohen’s *f*^2^ = 0.000). The interactions between RFGI and other demographical variables (age and income) were nonsignificant on the outcome variables (*ps* > 0.1).

Notably, there was a discrepancy between the results of gender-RFGI interplay on the two indicators of trust (amount sent and estimated among sent), implying that the moderation effect of gender on trust might not be very robust. These discrepant results between actual trust investment (with risk) and social expectations (without risk) could be attributed to women’s risk avoidance tendency [26], which causes them to act differently in situations with and without risk. This phenomenon also highlights the importance of measuring trust using both indicators in the investment game, as suggested by Ben-Ner et al. and Xin et al. [27, 39]. We further examined the moderation role of gender in Study 2.

### Study 2

#### Methods

##### Power analysis and participants

The sample size was estimated using G*Power 3.1.9.2. Given the Analysis of Covariance (ANCOVA) employed, as indicated by the software, the statistical test type was selected as “ANCOVA: fixed effects, main effects and interactions.” Following the lack of prior studies with identical designs, the effect size was set at the medium level for ANCOVA. Specifically, a medium effect size of 0.2526456 was chosen, which was converted from the medium effect size of partial η²=0.06. The alpha (α) level was set at 0.05, and the power was set at 0.95. The numerator *df* was determined through the software-prompted calculation formula as 1. To be specific, it was calculated as [2 (experiment conditions) -1] × [2 (the most categories in the nominal variables forming the interaction term; gender/origin) -1] = 1, with income and age as ordinal/continuous covariates. Based on these parameters, the required minimum sample size was calculated to be 206.

In this study, 456 university students were initially selected. 408 students provided valid responses (89.5%). The sample consisted of 212 males and 198 females. Among them, 154 came from urban backgrounds, while 254 were from rural areas. The academic distribution was as follows: 48 freshmen, 204 sophomores, 87 juniors, 58 seniors, 4 first-year graduate students, 7 second-year graduate students, and no third-year graduate students. Regarding monthly family income (in CNY), 115 students reported incomes below 3,000; 134 reported incomes between 3,000 and 5,000; 90 reported between 5,001 and 10,000. The average age was 20.826 years (*SD* = 2.665). Informed consent was formally obtained from all participants.

### Experimental design

The study employed a single-factor experimental design, comparing financial goal activation with a control group. The dependent variables were trust (indexed by investment amount in a trust game and estimated return rate) and trustworthiness (indexed by the return amount when participants acted as trustees).

### Materials and procedures

#### Financial goal activation and verification

Drawing on Bauer et al. 's prior research [23], we selected 24 internet images of luxury goods, such as high-end cars, clothing, and luxury hotels, to activate participants’ financial goals. Meanwhile, 24 landscape pictures from the same study were used for the control group. To conceal the true intent of the experiment, participants were asked to rate their pleasure from viewing each image on a scale from 0 to 7. To prevent repeated goal activation that could influence subsequent tasks, the efficacy of this manipulation was tested in a separate pilot study and not in the main experiment. In this pilot study, 62 students (26 males and 36 females) were randomly divided into either the activation group (*n* = 31) or the control group (*n* = 31). Using Teng et al.‘s [41] method for validating luxury image activation, a 2-item scale assessed the degree of materialistic activation. Participants indicated their agreement on a 7-point scale, and results demonstrated that the financial goal score of the activation group (*M* = 4.887, *SD* = 1.123) was significantly higher than that of the control group (*M* = 4.194, *SD* = 1.370) (*t* = 2.179, *p* = 0.033, Cohen’s *d* = 0.553), confirming the effectiveness of the manipulation.

### Trust and trustworthiness measurement

A similar investment game to that in Study 1 was conducted to assess trust and trustworthiness in this study. Trust was indicated by the among sent (0 to 100 CNY) and estimated return rate. In this study, we alternatively used a 7-point scale—0%(1), 17%(2), 33%(3), 50%(4), 67%(5), 83%(6). 100%(7) [42]—to assess how much (%) the participants expect the trustee would return (1 to 7). After that, participants played the role of a trustee, and they were asked how much (0 to 60) they would like to return if they received 60 CNY from a hypothetical trustor (who invested 20 CNY). It is worth noting that the participants’ received amount might affect their return rate (e.g., receiving more amount may cause a more favorable impression, thus giving more). Therefore, the different “received amounts” set in these two studies (90 CNY in Study 1 and 60 CNY in Study 2) can help evaluate the sensitivity of our results of the amount returned. The amount returned *M* = 32.745 out of 60 (54.6%) in Study 2 was comparable with that (*M* = 48.367 out of 90 [53.7%]) in Study 1. Therefore, the impact of the received amount is likely to be minimal.

### Awareness check

Participants were queried regarding their awareness of the study’s objectives and whether they perceived the activation’s effects on the subsequent trust game. Those who guessed the study’s aim or were aware of the manipulation’s impact were excluded.

### Procedures

The online experiment randomly assigned participants into either the activation or control groups. They were first introduced to the study as a “visual research task,” where they viewed and rated images. The activation group viewed 24 luxury images, while the control group viewed 24 landscape images. Participants then engaged in a “money distribution game” (investment game), after which they provided demographic information and underwent the awareness check. Following rule-checking and awareness verification processes, 48 participants (10.5%) were excluded due to a lack of significant difference between the groups (χ² = 0.701, *p* = 0.399).

## Results and discussion

The results of correlation analyses and descriptive statistics are shown in Table 3. Our findings of the trust (sent rate and estimated return rate) and trustworthiness (returned rate) (around 40%, 50%) are generally comparable with the previous findings in a meta-analysis [40].

**Table 3:** Descriptive statistics and correlation analysis (Study 2)

Variable	1	2	3	4	5	6	7	8	9
1. Gender	—								
2. Origin	-0.010	—							
3. Age	0.024	-0.035	—						
4. Income	0.055	0.196***	-0.035	—					
5. Experiment condition	0.175***	0.098*	-0.070	0.066	—				
6. Amount sent	0.042	0.038	0.112	0.102*	0.114*	—			
7. Estimated return rate	-0.003	0.110*	-0.068	0.169***	0.180***	0.468	—		
8. Amount returned	0.025	0.044	-0.056	0.021	0.110*	0.332	0.213^***^	—	
9. Pleasure	0.177	-0.005	0.009	0.061	0.133**	-0.045	-0.006	0.031	—
*Scaling*	0/1	0/1	—	1–9	0/1	0-100	1–7	0–60	1–7
*M*			20.826	2.512	—	44.123	3.735	32.745	5.053
*SD*			2.665	1.653	—	29.575	1.645	16.904	1.106

Note. ^*^ *p* < 0.05, ^**^ *p* < 0.01, ^***^ *p* < 0.001; unlike in Study 1, the estimated return rate in Study 2 was measured by a 7-point scale—0%(1), 17%(2), 33%(3), 50%(4), 67%(5), 83%(6). 100%(7) [33]

**Fig. 1:**
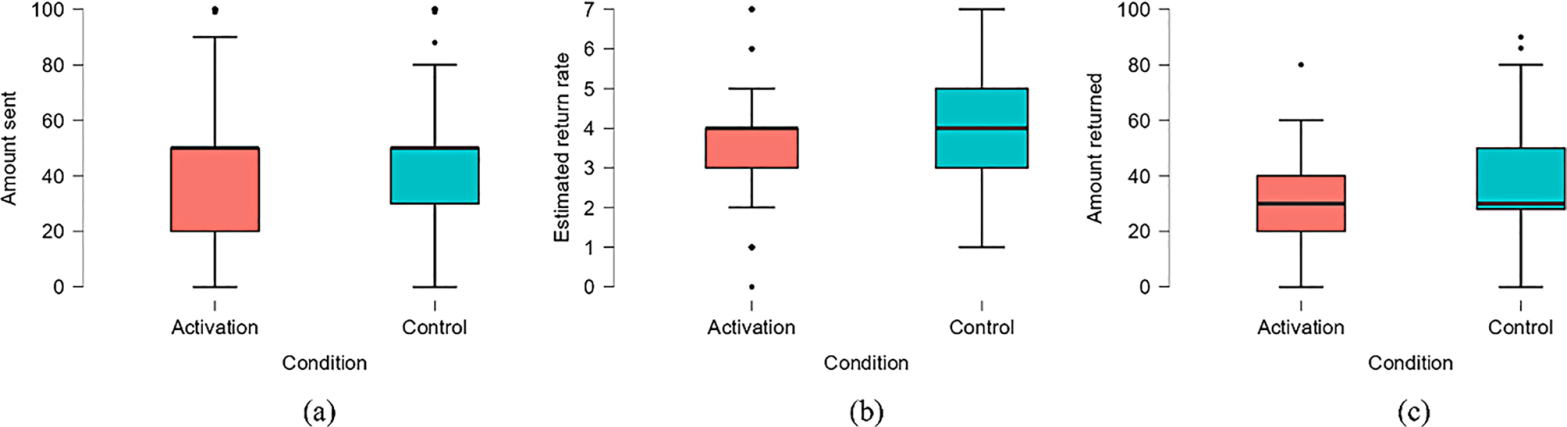
Amount sent (**a**), estimated return rate (**b**), amount returned (**c**) in different experimental conditions (financial goal activation condition versus control condition)

We assessed the impact of experimental manipulation on participants’ trust and perceived trustworthiness, along with the interaction effects between experimental conditions and demographic variables, using a Multi-way Analysis of Covariance (ANCOVA). The categorical independent variables, namely experimental conditions (activation and control), gender, and place of origin, were treated as factors. The continuous/ordinal independent variables, such as age, income, and pleasure elicited by the experimental manipulation, were included as covariates. Interaction terms were constructed between the experimental conditions and other independent variables. The dependent variables were the amount sent and estimated return rate by the participants in the investment game when acting as the trustor and the amount returned when acting as the trustee. The amount sent, estimated return rate, and amount returned for participants across different experimental conditions are presented in Table 4; Fig. 1.

**Table 4:** Amounts sent, estimated return rate, and amount returned under different experimental conditions

Outcome	Condition	*n*	M	SD
Amount sent	Activation	219	41.000	27.788
	Control	189	47.741	31.205
Estimated return rate	Activation	219	3.461	1.485
	Control	189	4.053	1.765
Amount returned	Activation	219	31.023	15.976
	Control	189	34.741	17.754

The experimental condition’s main effects on the amount sent, estimated return rate, and amount returned, as well as its interaction effects with gender and origin, are detailed in Table 5. It’s important to note that interactions that were not significant—specifically, those between the experimental condition and age, income, and the pleasure derived from the experimental manipulation, along with the interactions between the experimental condition and both gender and origin regarding the amount returned (*p*s > 0.1)—have been omitted from the analysis for simplicity.

As shown in Table 5, the experimental condition’s negative main effects significantly influenced trust—as indexed by the amount sent and the estimated return rate—and trustworthiness, as indicated by the amount returned (*p*s < 0.05). Trust and trustworthiness of participants in the financial goal activation condition were significantly lower than that in the control condition, indicating that situational financial goal can significantly reduce participants’ trust and trustworthiness.

**Fig. 2:**
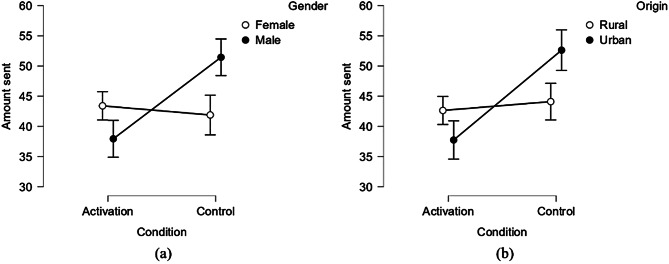
The moderation roles of gender (**a**) and origin (**b**) of college students for amount sent

Additionally, significant interactions between the experimental condition and both gender and origin were observed in terms of the amount sent (*p*s < 0.05). The amount sent by participants with different genders and origins in different conditions are shown in Table 6. The results of simple effect analyses are in Fig. 2. The negative effect of the experimental condition on amount sent was significant among male students (*F*_1, 205_ = 11.514, *p* = 0.001, *η²* = 0.053) but not among female students (*F*_1, 189_ = 0.056, *p* = 0.813, *η²* = 0.000), and it was significant among urban students (*F*_1, 147_ = 8.513, *p* = 0.004, *η²* = 0.055) while not significant among rural students (*F*_1, 247_ = 0.228, *p* = 0.634, *η²* = 0.001).

**Table 5:** ANCOVA with interactions (Study 2)

	Amount sent			Estimated return rate			Amount returned		
	*F*_(1, 399)_	*p*	*η²*	*F*_(1, 399)_	*p*	*η²*	*F*_(1, 401)_	*p*	*η²*
Condition	7.159	0.008	0.017	12.571	< 0.001	0.029	3.814	0.052	0.009
Gender	0.337	0.562	0.001	0.232	0.630	0.000	0.012	0.911	0.000
Origin	0.016	0.900	0.000	1.188	0.276	0.003	0.391	0.532	0.000
Condition*gender	5.267	0.022	0.012	10.750	0.001	0.025	—	—	—
Condition*origin	4.190	0.041	0.010	2.732	0.099	0.006	—	—	—
Age	5.023	0.026	0.012	1.999	0.158	0.005	0.938	0.333	0.000
Income	3.282	0.071	0.008	8.216	0.004	0.019	0.011	0.917	0.000
Pleasure	2.039	0.154	0.005	0.379	0.538	0.000	0.117	0.733	0.000

Note. The nonsignificant interactions of condition with age and income were excluded for simplicity

**Table 6:** Amount sent and estimated return rate by gender and origin in different experimental conditions

	Moderator and category	*n*	M	SD
**Amount sent**				
Condition	Gender			
Activation	Female	123	43.390	25.858
	Male	96	37.938	29.939
Control	Female	73	41.877	28.036
	Male	116	51.431	32.621
Condition	Origin			
Activation	Rural	146	42.630	28.121
	Urban	73	37.740	27.003
Control	Rural	108	44.093	31.490
	Urban	81	52.605	30.333
**Estimated return rate**				
Condition	Gender			
Activation	Female	123	3.715	1.417
	Male	96	3.135	1.512
Control	Female	73	3.781	1.609
	Male	116	4.224	1.842
Condition	Origin			
Activation	Rural	146	3.452	1.453
	Urban	73	3.479	1.556
Control	Rural	108	3.787	1.669
	Urban	81	4.407	1.836

The interaction between condition and gender on estimated return rate was significant (*p* = 0.001). The effect of the experimental condition was significant among male students (*F*_1, 205_ = 26.418, *p* < 0.001, *η²* = 0.114), while it became nonsignificant among female students (*F*_1, 189_ = 0.084, *p* = 0.774, *η²* = 0.000). However, the interaction between the experimental condition and origin did not reach traditional levels of significance (*p* = 0.099). It is crucial to recognize that the conventional threshold of *p* < 0.05 is somewhat arbitrary and subject to ongoing debate. A *p*-value of 0.099 can be evidence of “marginal” or “suggestive” significance, meaning the interaction between condition and origin on estimated return rate could potentially be meaningful. Thus, we still looked into its interaction pattern.

**Fig. 3:**
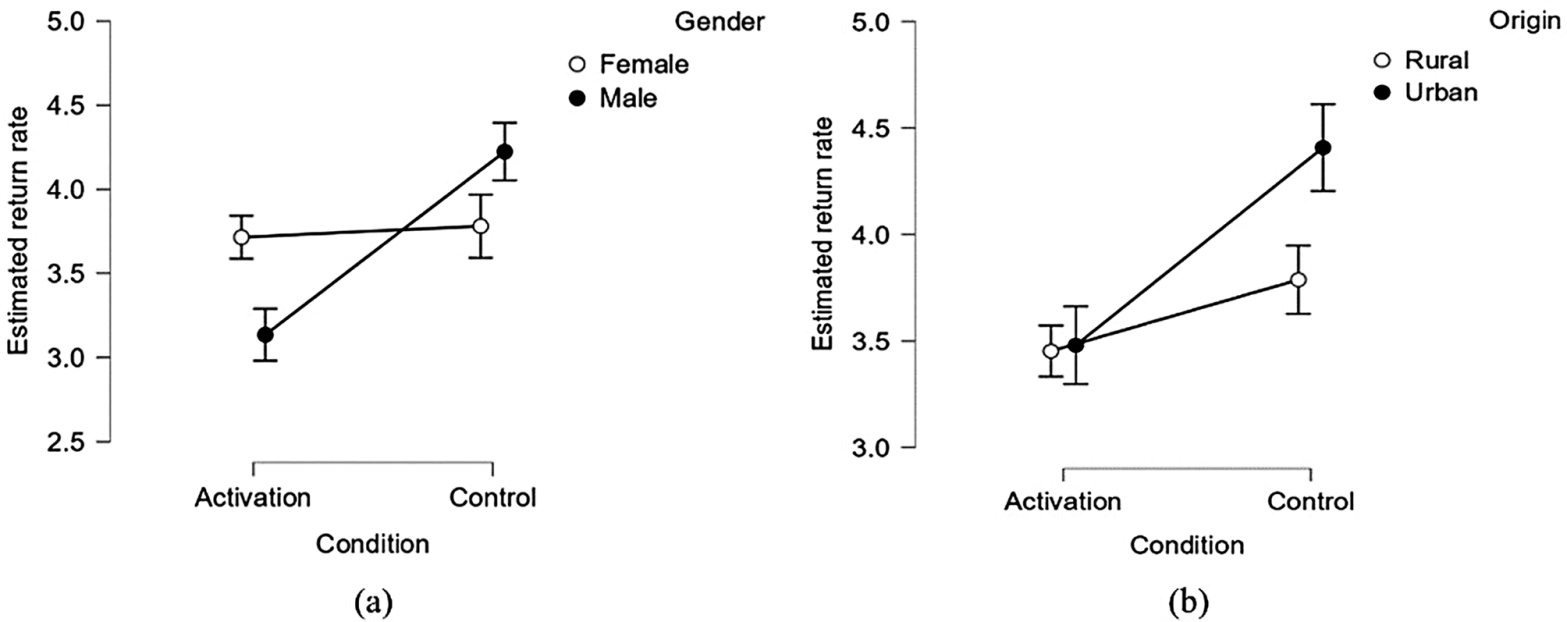
The moderation roles of gender (**a**) and origin (**b**) of college students for the estimated return rate

Table 6 displays the estimated return rate for participants across various genders and origins under different experimental conditions, with Fig. 3 illustrating the corresponding interaction patterns. The results of simple effect analyses showed that the effect of condition on estimated return rate among urban students (*F*_1, 147_ = 8.895, *p* = 0.003, *η²* = 0.057) was larger than that among rural students (*F*_1,247_ = 2.777, *p* = 0.097, *η²* = 0.011), similar with the interaction pattern of condition and origin on amount sent.

Notably, the results of the interplay between financial goal activation and gender on trust in Study 2 are slightly different from Study 1 regarding its effect on amount sent. That said, the role of gender was generally similar on most of occasions (except for its moderation effect for amount sent in Study 1).

Interactions involving the experimental condition, gender, and origin had no significant effect on the amount returned, suggesting that, unlike in Study 1, gender and origin do not modulate the impact of situational financial goals on trustworthiness. This stability might stem from the perception of trustworthiness—as opposed to trust—as a universal ethical standard within societal norms, suggesting it remains consistent across different scenarios. This may also be why trustworthiness was altered by experimental manipulation to a relatively small extent that only reached marginal significance (compared to the prominent prediction by dispositional financial goal pursuit in Study 1). Thus, the contribution of the interactions between financial goal activation and the moderators on the trustworthiness change might not be detected due to its relatively small overall alteration; namely, students, regardless of their genders or origins, tend to change relatively little from their original levels of trustworthiness when their financial goal pursuit is activated by a temporary situation, thus failing to form significant interactions.

### General discussion

The present research reveals a negative impact of financial goals on trust and trustworthiness, thereby expanding the existing literature on the aftermath of financial goals [43, 44]. Moreover, by employing a survey and an experiment, our study provides more direct evidence of the causal relationship between financial goals and trust and trustworthiness. The experimental method confirms our results alongside the alignment with previous survey-based correlational studies, such as that by Li [45], and is consistent with our theoretical speculations based on indirect evidence [46, 47]. Importantly, the sample for our study is drawn from collectivist China rather than individualistic Western countries, a rarity in the literature that nonetheless yielded similar conclusions. This similarity may be attributed to the global rise of individualism and the decline of collectivism, especially among younger populations [48], suggesting that the pursuit of financial goals, a typically individualistic value, is becoming more prevalent even in collectivist countries. Therefore, this study examines the relationship between financial goals and trust in a Chinese context, reaching conclusions that corroborate those from different cultural backgrounds and diminish the potential impact of cultural values on these dynamics. This provides a new perspective for understanding the paradox of China’s recent economic boom, which was accompanied by a decline in interpersonal trust. The findings of this study support the hypothesis that the pursuit of financial goals is a significant factor in diminishing interpersonal trust, illustrating that the advancement of marketization and the growth of the market economy also led to the excessive pursuit of material wealth. As material benefits or money become primary goals for individuals, they are more inclined to risk losing trust.

This finding aligns with previous research on materialism and interpersonal relationships. Existing literature posits that materialism, characterized by an emphasis on wealth and possessions, can compromise prosocial behaviors and interpersonal trust [22, 23, 46, 47]. Individuals prioritizing financial success may tend to favor self-interest over cooperative behavior, thereby eroding trust and trustworthiness.

This suggests that developing countries, including China, should be cautious of the potential negative consequences of the marketization process. Governments and researchers should implement measures such as establishing reliable market regulations to mitigate possible psychological costs. However, it’s important to exercise caution when extending this implication to other developed countries, as people in wealthier nations, with more opportunities and greater purchasing power, are less likely to consider wealth as the main goal of life [49]; thus, the demographics holding financial goals in developed countries are likely to be significantly different from those in developing countries and should not be conflated. Future research could involve sampling in developed countries for a comparative study to gain a more comprehensive understanding of the issue.

Additionally, this research indicates that financial goal pursuit’s detrimental effects on trust (in Studies 1 and 2) and trustworthiness (in Study 1) are more pronounced among male college students and those from urban areas compared to their female and rural counterparts.

This gender disparity pattern does not support the predictions made based on parental investment theory and social role theory [26]. Conversely, this finding aligns with recent research exploring the interplay of gender, competition, and societal values. One possible explanation for this gender difference lies in the societal expectations and socialization processes that males and females undergo, which may shape their attitudes toward financial success and its implications for trust and trustworthiness. It is commonly observed that males are often socialized to be more competitive and oriented towards achievement [50]. This competitive orientation could lead to a greater emphasis on personal gain and a suspicious perception of others [22], deteriorating the negative effect of financial goal pursuit on cooperative behaviors and interpersonal trust among men. In contrast, women, for instance, have been found to place more emphasis on relational and communal values, which could potentially buffer the negative influence of financial goals on trust [2, 51].

Drawing upon Dittmar’s consumer culture impact model [31], the urban-rural disparity in the impact of financial goal pursuit on trust may resonate with the stark socioeconomic differences between China’s urban and rural regions [52]. Rapid urbanization, coupled with increased competition, consumerism, and individualism, may contribute to the stronger effect observed among urban students. Conversely, rural areas, potentially characterized by stronger community ties and less pronounced consumerism, may be more insulated from the negative effects of financial goal pursuit.

We found that age and family income did not moderate the negative impact of financial goal pursuit on trust and trustworthiness. This indicates that, among college students, high financial goal pursuit likely consistently weakens trust and trustworthiness, regardless of age and family income. This aligns with prior studies showing a negative link between financial goal pursuit and trust/ trustworthiness [22, 23], but contradicts findings that age and family income might alter trust or trustworthiness [32–34]. This inconsistency may stem from sample characteristic differences, as college students have more homogeneous ages and incomes than working adults, limiting significant findings. For instance, comparing high-income, middle-income, and low-income working adult groups might show low- income groups experience stronger negative effects. Similarly, comparing youth, middle-aged, and elderly groups might find youth are more affected. Future research could consider sampling diverse populations with wider-ranging characteristics to better reveal individual and group differences in this context.

It is worth noting that the discrepancies in results between the two trust indicators (in Study 1) and the results for trustworthiness between Study 1 and Study 2 imply that more studies are warranted. These studies should further test the robustness of our findings on the moderation effects, and the interpretation and generalization of these findings should be approached with caution.

Our findings provide evidence of the relationship between financial goal pursuit, trust, and trustworthiness in a Chinese context. They highlight the need for more nuanced research to unravel the interplay between economic aspirations, sociocultural factors, and interpersonal relationships in China. Furthermore, they emphasize the need for fostering balanced societal development, one that promotes economic growth while simultaneously encouraging interpersonal trust and social cohesion.

The findings on the moderators in the links between financial goal pursuit and trust and trustworthiness also contribute to a growing body of literature on gender differences in economic behavior and their repercussions on social outcomes. They underscore the need for a gender-sensitive approach in studying the effects of financial goal pursuit, highlighting the importance of considering how gendered socialization and societal norms might interact with economic aspirations to shape trust and trustworthiness. Furthermore, these findings gain relevance when viewed against the backdrop of China’s rapid economic transformation and urbanization. The importance of financial success has been amplified in recent decades due to economic modernization and the shift toward a market-oriented economy [2]. This societal shift may have profound impacts on social relationships, especially among males and urban dwellers, who appear more susceptible to the negative effects of financial goals on trust.

For educators and university administrators, our findings suggest that attention should be paid to the financial goal pursuits of university students, especially among male students and those from urban areas. Specifically, it is necessary to carefully consider the potential repercussions that emphasizing financial success might have in the educational environment. Some universities in China tend to promote their funding or scholarship schemes by posting advertising at the most visible locations on campus, which could be a situational clue activating financial aspirations. While it is essential for universities to advertise and communicate about funding and scholarship opportunities, they can try to balance this with messages that promote other forms of success and values. This could include promoting academic contributions, community involvement, creative endeavors, and values such as kindness, teamwork, and empathy. Universities can consider launching campaigns that celebrate these aspects to create a more balanced perception of success. By promoting a balanced perspective and fostering an environment that values trust and cooperation, it may be possible to mitigate some of the negative effects associated with the pursuit of financial success.

This research has several limitations: First, the generalizability of the findings may be limited due to the specific demographic chosen for this study, as we focused only on Chinese university students, resulting in a somewhat homogeneous sample; some novel results might not be applicable to other cultural or age groups. Future studies could broaden this line of inquiry to include diverse populations, aiming to ascertain if these results are consistent across different cultural or socioeconomic contexts. Second, the study did not delve into the potential psychological or cognitive mechanisms that underlie the observed associations between financial goals and trust and trustworthiness. Future research can explore the potential mediating variables that might account for this relationship. For instance, could competitive tendencies mediate this relationship? Third, the study adopted the hypothetical version of the investment game as the measure of trust and trustworthiness. While hypothetical game tasks have been used in previous studies and have been regarded as almost equivalent to the game tasks with actual monetary income [27, 38, 53], future research can further examine our findings using actual game tasks or other measures for trust and trustworthiness assessment. Fourth, apart from the control variables in this study, the literature provides a number of factors related to interpersonal trust, such as personality traits, self-esteem, and academic pursuits (i.e., a student’s major). For incidence, students majoring in economic disciplines may exhibit lower levels of trust compared to those in other majors [54]. Nonetheless, it could be impractical for a single study to cover the full spectrum of related variables. Future research is encouraged to encompass a broader array of covariates and explore their potential interactions with financial goals in predicting trust/trustworthiness, deepening our understanding of these relationships. For example, investigating how studying economic disciplines could shape financial goals, which in turn affects interpersonal trust (a mediation mechanism). Last, we exclusively focused on generalized trust but not particularistic trust (i.e., trust in specific targets such as friends, teachers, journalists, etc.). Future research can explore the impacts of financial goal pursuit on particularistic trust.

## Conclusion

This research examined the influence of financial goal pursuit on trust and trustworthiness and the moderating roles of demographical variables among Chinese college students via two studies. Drawing upon value system theories, we proposed hypotheses that financial goal pursuit undermines trust and trustworthiness. Our results support these hypotheses. Meanwhile, gender and origin are two significant moderators that alter the influences of financial goal pursuit. Specifically, the negative effects of financial goal pursuit on trust (Study 1 and 2) and trustworthiness (Study 1) are more prominent among male students (than female students) and urban students (than those from rural areas), while age, family monthly income, and manipulation-induced pleasure cannot moderate the influences of financial goal pursuit.

## Data Availability

The datasets generated and analyzed during the current study are not publicly available but are available from the corresponding author upon reasonable request.

## Abbreviations

AI–6: Aspiration Index-6 items
RFGI: Relative Financial Goal Importance
ANOVA: Analysis of Variance
ANCOVA: Analysis of Covariance
